# Coping with emotions in pandemic as a factor of somatic complaints during lockdown

**DOI:** 10.1192/j.eurpsy.2022.1247

**Published:** 2022-09-01

**Authors:** Y. Chernetsova, V. Emelin, E. Rasskazova, A. Tkhostov

**Affiliations:** 1 Moscow State University, Clinical Psychology, Mokhovaja, Russian Federation; 2 Mental Health Research Center, Medical Psychology, Moscow, Russian Federation; 3 Moscow State University, Clinical Psychology, Moscow, Russian Federation

**Keywords:** lockdown, expression of emotions, somatic complaints, affective complaints

## Abstract

**Introduction:**

Increase in affective and somatic complaints during pandemic is considered as related to experienced stress (Wang et al., 2020, Roy et al., 2020, Robillard et al., 2020). Expression or suppression of emotions related to pandemic could affect the vulnerability of people to stressful situations (Gross, Thompson, 2007, Roberts et al., 2008).

**Objectives:**

The aim was to reveal a role of suppression / expression emotions regarding pandemic in the changes in somatic and affective complaints in people without coronavirus during lockdown.

**Methods:**

In May 2020 110 people 18-65 years old (61.2% females) without coronavirus appraised their strategy of dealing with different emotions regarding pandemic on the 1-5 scale from emotional expression to hiding and suppression (Cronbach’s alphas) and 26 somatic and emotional symptoms including sleep-related symptoms, daytime functioning, affective symptoms, general physical condition (Cronbach’s alphas .81-.90). In December 2020 they reappraised 26 complaints.

**Results:**

There were no statistically significant changes in somatic and affective complaints during May-December 2020 (p>.20). Increase in sleep-related complaints (β=.23, p<.05, ΔR^2^=5.0%) and complaints regarding general physical condition (β=.32, p<.05, ΔR^2^=10.0%) were more pronounced in those reporting higher expression of emotions related to COVID.

**Conclusions:**

People with higher emotional reactivity to pandemic situation tend to report increase in sleep-related problems and general worsening of their physical condition during lockdown. Research is supported by the Russian Foundation for Basic Research, project No. 20-013-00799.

**Disclosure:**

Research is supported by the Russian Foundation for Basic Research, project No. 20-013-00799

